# Decision Rules in Frequentist and Bayesian Hypothesis Testing: P-Value and Bayes Factor

**DOI:** 10.3389/ijph.2025.1608258

**Published:** 2025-05-14

**Authors:** Mario Fordellone, Paola Schiattarella, Giovanni Nicolao, Simona Signoriello, Paolo Chiodini

**Affiliations:** Unità di Statistica Medica, Dipartimento di Salute Mentale e Fisica e Medicina Preventiva, Università degli Studi della Campania Luigi Vanvitelli, Naples, Italy

**Keywords:** bayes factor, p-value, hypothesis testing, bayesian analysis, bayesian approach

## The Philosophy of the P-value

The p-value, a landmark statistical tool dating from the 18th century, remains a widely used measure in inferential statistics, representing the probability of obtaining a result at least as extreme as the observed one, given that the null hypothesis ( H0 ) is true [1–4]. It operates under the assumption that H0 holds but doesn’t directly assess the validity of the null hypothesis or the likelihood that the observed results occurred by chance [5]. One of its major advantages is that its interpretation is intuitive: the smaller the p-value, the less likely it is that the observed results are compatible with the null hypothesis [6].

However, the p-value has significant limitations. For instance, p-value is sensitive to the sample size. By increasing the sample size, the power of the test increases. Therefore, in very large samples, even minor and clinically irrelevant effects can yield statistically significant p-values, while important effects might go undetected in smaller samples [1].

Alternatively, for a wide range of statistical tests, lowering the significance threshold reduces the chance of false positives, but would also require an increase in sample sizes to maintain the same power [7].

Moreover, relying on a fixed threshold to determine significance can lead to binary interpretations of results (significant vs. not significant) that fail to capture the continuum of statistical evidence. This challenge led researchers to integrate the analyses with additional metrics, such as confidence intervals, that provide a range of values derived from the sample data within which the population value is likely to fall [8–11].

Lastly, the p-value itself provides no information regarding the evidence in favor of an alternative hypothesis. While a small p-value, according to confidence intervals, may suggest that the data do not support H0 , it fails to quantify from a comparative perspective how much more likely the data are under an alternative hypothesis H1 , leaving researchers without a clear measure of relative evidence between the hypotheses [12].

Widespread misusages concerning the p-value encourage statisticians to explore alternative approaches, such as the Bayes Factor [13]. For further insights on the limitations and misconceptions about the p-value, see also [14–17].

## Understanding Bayes-Factor

The Bayesian approach to hypothesis testing was developed by Jeffreys in 1935 [18, 19]. The method, now referred to as Bayes Factor (BF), is a Bayesian tool used to compare the evidence in favor of two hypotheses. It compares the likelihood of the data under the null hypothesis H0 to the likelihood under the alternative hypothesis H1 . Therefore, unlike the p-value, the BF directly measures how likely the data are under each hypothesis, providing a quantitative comparison between H0 and H1 [12].

The BF converts prior odds, that represent the ratio of the initial probabilities assigned to the two hypotheses before observing the data, to posterior odds by incorporating the data ( y ). Formally, the BF can be defined as the ratio of the probability of observing the data given H1 and the probability of observing the data given H0 .

$$\underset{\begin{array}{c} Posterior\;\\ \text{odds} \end{array}}{\underbrace{\frac{P\left(\left.H_{1}\right|y\right)}{P\left(H_{0}\left|y\right.\right)}}}=\underset{Bayes\;Factor}{\underbrace{\frac{P\left(\left.y\right|H_{1}\right)}{P\left(\left.y\right|H_{0}\right)}}}\times \underset{\begin{array}{c} \text{Prior}\\ \text{odds} \end{array}}{\underbrace{\frac{P\left(H_{1}\right)}{P\left(H_{0}\right)}}}.$$ (1)

Several categorizations were proposed in the form of ratio and compared [12, 18, 20–22]. By considering Formula 1, the BF value can be interpreted as shown in Table 1.

**TABLE 1:** Guidelines for interpreting the bayes factor (Naples, Italy. 2025).

BF value^a^	Interpretation
<0.01	strong to very strong evidence for H_0_
0.01–0.03	strong evidence for H_0_
0.03–0.1	moderate to strong evidence for H_0_
0.1–0.33	weak to moderate evidence for H_0_
0.33–1	negligible evidence for H_0_
1	no evidence
1–3	negligible evidence for H_1_
3–10	weak to moderate evidence for H_1_
10–30	moderate to strong evidence for H_1_
30–100	strong evidence for H_1_
>100	strong to very strong evidence for H_1_

^a^ The researcher should be aware that this scale applies when *H*_*1*_ is in the numerator.

One notable advantage of the BF is its ability to provide a continuous measure of evidence supporting or opposing a hypothesis and its values varies, from strong support for H0 to strong support for H1 [21].

Another benefit is that the BF allows the incorporation of prior information, such as pre-existing knowledge or theoretical assumptions into the analyses, enhancing the robustness of the results.

The data-based BF finds a critical limitation in its sensitivity to the prior choice [21]. Therefore, it is crucial to set priors on a solid pre-existing knowledge or to select them in a conservative way [18]. Alternative methodological approaches to the BF are discussed in [23–26].

## Comparing P-Value and Bayes-Factor: A Simulation Study

In literature, many authors focus their research on the comparative study of p-value and BF. Reader can refer to a brief literature review provided in the Supplementary Material [21, 27–35]. Moreover, BF is implemented in various R packages, which offer diverse functionalities for their computation [36–39].

### Simulation Design

The simulation proposed in this work was designed to evaluate the behavior of the p-value and the BF in a two-sample t-test comparing the means of two groups. Comprehensive details on how the simulation was conducted are included in the Supplementary Material.

### Results

**FIGURE 1:**
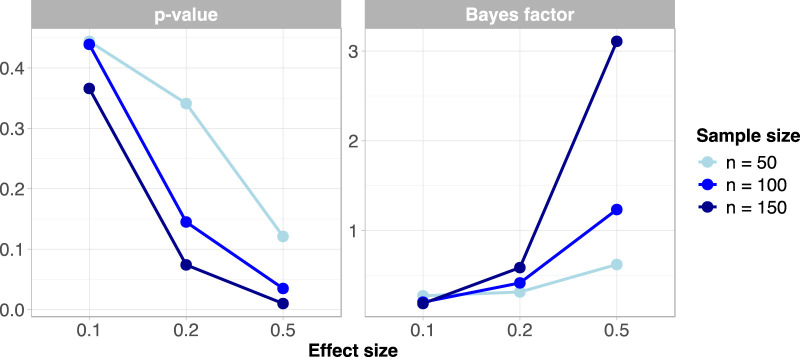
Comparing results between p-value and Bayes factor in the simulation study (Naples, Italy. 2025).

Figure 1 showed the comparative results between p-value and BF in the simulation study. In particular, the medians of p-value and BF simulated distributions were reported. In general, the BF is less sensitive to sample size in the presence of mild effects of 0.1 and 0.2. It can also be observed that the p-value takes an extremely low value in the presence of an effect of 0.5 for a sample size of 150, meanwhile the BF is more cautious since it supports moderate evidence in favor of the alternative hypothesis. Moreover, when the effect size is at 0.5 and n is 100, the p-value corroborates the rejection of the null hypothesis, while the evidence for H1 from the BF is barely worth mentioning. However, the p-value is sensitive to sample size only when the null hypothesis is false, while BF seems to be affected by sample size both in the presence and absence of true effects.

## Concluding Remarks

This paper presents a comparison between p-value and BF in hypothesis testing, accompanied by a concise literature review on the subject. Findings from our simulation study align with existing literature, revealing that p-values are more sensitive to variations in sample size and effect size compared to BF. Moreover, BF provide a more nuanced approach to decision-making, offering flexibility beyond the binary accept/reject framework of the null hypothesis. Nevertheless, a controversial aspect is that BF are sensitive to the choice of prior distribution, which can decisively impact the results, especially in more complex settings where researchers must be particularly careful in their implementation.
