# Supplementary material for: Decision Rules in Frequentist and Bayesian Hypothesis Testing: P-Value and Bayes Factor
Source: Int J Public Health. 2025 May 14;70:1608258. doi: 10.3389/ijph.2025.1608258 (PMC12123088; doi:10.3389/ijph.2025.1608258)
Supplement: Supplementary file 1 [file DataSheet1.docx]

**Decision Rules in frequentist and Bayesian Hypothesis testing: p-Value and Bayes Factor**

[Supplementary materials]

1. **Comparing p-value and Bayes Factor: rationale and a brief literature review**

In this section a brief literature review on the comparative studies of p-value and BF was provided. In fact, many authors focus their research on this topic. For example, [27] aim to give readers an understanding of Bayesian methods, including the Bayes factor (BF) in the context of hypothesis testing, in comparison to traditional approaches such as the p-value, and present several practical examples, while acknowledging its usefulness in addressing problems in the health sciences.

[28] provides a critical perspective, arguing that p-values can encourage overly simplified inference in the epidemiologic field. The author suggests the minimum BF as an alternative that could provide a different view of results and discussion sections. [29] investigated the implications of nominal statistical significance on the credibility of null versus alternative hypotheses across a wide range of observational epidemiologic associations that claimed formal statistical significance (p < 0.05), finding that these associations provided weak support when evaluated using the BF. [30] conducted their research within the field of genetic epidemiology and propose an alternative method for calculating the BF. Their contribution includes establishing a link between BF and p-values through a specific prior, with particular emphasis on effect size. In [31] the case of the independent samples t-test was considered, where the p-value and BF were evaluated through a Monte Carlo study across different sample sizes and effect sizes. The greatest discrepancies occur in cases of low and high sample sizes and in those with a small effect size. [32] perform a comparison between the p-value and posterior probability in a clinical trial. The results show that, even when rejecting the null hypothesis, a probability around 20% that the null hypothesis is true remained, emphasizing that the posterior probability and the BF could provide complementary evidence. [33] criticize the automatic interpretation of BFs while suggesting to avoid a binary view of inference. They recommend focusing on effect sizes and intervals rather than binary decisions. The BF should be viewed as a measure of evidence, reflecting the flexibility of Bayesian philosophy, encouraging interpretations based on the analyst's prior knowledge. [34] highlight some of the misconceptions about the p-value and illustrate its performance using some simulated experiments, setting different prior choices. [21] provide a review of the available literature considering two-sided significance tests for a point null hypothesis in more detail and focusing mainly on the minimum BF and its relationship with the p-value. [35] examine recent proposals to integrate or modify the p-value, following the ASA statement of 2016 from a Bayesian perspective by analyzing two case studies on pharmacotherapy in infectious diseases. The authors analyze the BF within the first section, suggest abandoning fixed thresholds and urge statistical societies to promote new perspectives.

In the field of health statistics, the p-value remains an essential tool, but it needs to be read carefully due to its binary nature. Training researchers in alternative methodologies that enhance more comprehensive assessments, such as the BF, and their introduction in the analyses may improve the quality of scientific evidence and research.

However, it is appropriate to consider the limitations related to the comparison between the p-value and the BF. While the former follows a frequentist approach, the latter operates within the Bayesian framework, which is intrinsically subjectivist and based on a different probabilistic system, that incorporates prior information into the analysis, forming the a priori distribution. For the frequentist framework, most argument are used for a p-value with a specific decision threshold, while the Bayes factor is interpreted mostly continuously. Thus, the comparison conflates the frequentist-vs-Bayesian dichotomy with the binary-vs-continuous "decision" one, making it difficult to draw sound conclusion. Moreover, some interpret p-values continuously (e.g., see Fisher's interpretation as the strength of evidence against the null hypothesis) and that for binary decisions selecting a threshold becomes necessary also for Bayes factors.

1. **Simulation scheme**

Nine distinct scenarios were generated by combining three levels of effect size, defined as the standardized difference between the two group means (i.e., 0.1, 0.2, and 0.5), and three different sample sizes for each of the two groups (i.e., 50, 100, and 150). For each scenario, the simulation was repeated 5000 times. This approach allows for a clear comparison of the two inferential methods in terms of their sensitivity to effect size and sample size. For BF a prior on Cohen's d (the standardized effect size) for the alternative hypothesis was applied. The default prior is a Cauchy distribution centered at 0 and was moderately informative. The use of a single prior was intended for illustrative purpose, providing a practical demonstration of the behavior of the BF in hypothesis testing, while acknowledging that varying the parameters of the prior could offer additional insights into the BF’s behavior depending on effect size and sample size. Moreover, this application should not be interpreted as a general recommendation for all cases. The researcher should note that selecting an appropriate prior distribution is not a trivial task and requires careful consideration based on domain knowledge and the specific research context. The simulation was conducted using the R package BayesFactor [36].

A more comprehensive analysis would require comparing additional types of tests and providing stronger demonstrations of the sensitivity to sample size in both approaches, and this could be a limitation of this simulation study. However, the primary goal was to illustrate, in an intuitive manner, how p-value and BF respond differently to varying sample size and effect size.

**R code**

# Require packages

library(readxl)

library(BayesFactor)

library(ggplot2)

library(writexl)

# Parameters

n_samples = xx # Sample size

mu_H0 = 0 # Effect under H0

low = 0.1 # Effect under H1

moderate = 0.2 # Effect under H1

high = 0.5 # Effect under H1

sigma = 1 # Standard deviation

n_simulations = 5000 # Simulation runs

# Initialize p-value e Bayes Factors vectors

pvalue <- numeric(n_simulations)

bayes_factors <- numeric(n_simulations)

# Simulations

for (i in 1:n_simulations) {

data_H0 <- rnorm(n_samples, mean = mu_H0, sd = sigma)

data_H1 <- rnorm(n_samples, mean = xx, sd = sigma)

t_test_result <- t.test(data_H0, data_H1)

pvalue[i] <- t_test_result$p.value

combined_data <- c(data_H0, data_H1)

group <- factor(c(rep(1, n_samples), rep(2, n_samples)))

ddata <- cbind.data.frame("value" = combined_data,

"group" = group)

bf_result <- ttestBF(formula = value ~ group, data = ddata)

bayes_factors[i] <- exp(bf_result@bayesFactor$bf)

print(paste("Iteration n.", i))

}

median(x = pvalue)

median(x = bayes_factors)

dd <- cbind.data.frame(pvalue, bayes_factors)

write_xlsx(x = dd, path = "dd.xlsx")

***Figure S1 – p-value distributions on 5000 random starts for different effect size and sample size scenarios. Panel (a): sample size equal to 50; Panel (b): sample size equal to 100; Panel (c): sample size equal to 150.***

| 1. Sample size equal to 50    | 1. Sample size equal to 100    |
| --- | --- |
|  |  |
| 1. Sample size equal to 150    |  |

***Figure S2 – Bayes Factor distributions on 5000 random starts for different effect size and sample size scenarios. Panel (a): sample size equal to 50; Panel (b): sample size equal to 100; Panel (c): sample size equal to 150.***

| 1. Sample size equal to 50    | 1. Sample size equal to 100    |
| --- | --- |
| 1. Sample size equal to 150    |  |
